# Late Isolated Central Nervous System Relapse from Ovarian Serous Adenocarcinoma: A Case Report and Literature Review

**DOI:** 10.1155/2014/297307

**Published:** 2014-11-18

**Authors:** Tiago Biachi de Castria, Sylvia Regina Quintanilha Rodrigues, Maria del Pilar Estevez Diz

**Affiliations:** ^1^Department of Medical Oncology, Instituto do Cancer do Estado de Sao Paulo, 251 Doutor Arnaldo Avenue, 01246-000 Sao Paulo, SP, Brazil; ^2^Faculdade de Medicina, Universidade de Sao Paulo, 455 Doutor Arnaldo Avenue, 01246-000 Sao Paulo, SP, Brazil

## Abstract

Central nervous system involvement by ovarian serous adenocarcinoma is rare. We report a case of a 60-year-old woman that developed brain metastasis as isolated site of relapse 4.5 years after a complete resection and adjuvant chemotherapy for a stage Ic disease. She proceeded to a craniotomy with resection of the lesion and, subsequently, to a whole brain radiotherapy. Nineteen months later, she developed carcinomatous meningitis as isolated site of recurrence. Patient was submitted to intrathecal chemotherapy with methotrexate; however, she died from progressive neurologic involvement disease few weeks later.

## 1. Introduction

Ovarian cancer is the most common gynecologic cancer in United States: in 2014, 24,000 new cases and 14,000 deaths are expected [1]. Worldwide 240,000 new cases and 150,000 deaths were expected in 2012 [2]. Despite being a rare disease when compared to other tumors and treatment improvement with chemotherapy, mortality from ovarian cancer has remained high over the past 4 decades (5-year survival rate 33% in 1975 and 45% in 2006) [1].

Late diagnosis and recurrence are common in epithelial ovarian cancer (EOC); however, central nervous system (CNS) is the site of recurrence in only 1-2% of the cases [3, 4]. Usually, relapses occur through peritoneal or lymphatic spread and rarely hematogenous dissemination beyond abdominal caveat is present. Among patients who developed isolated brain metastases (BM), 75% occur in advanced tumors (International Federation of Gynecology and Obstetrics, FIGO III-IV), which can be explained by the control of abdominal disease by current regimens of chemotherapy with low CNS penetration [3].

Since BM from EOC is a rare condition, medical knowledge is limited to case reports or series of patients and less than 600 cases are documented to date in the literature [5].

Once systemic treatment is improving survival in metastatic patients and, probably, treating with more efficacy micrometastatic disease in adjuvant scenario, CNS metastasis (CNSm) from EOC is likely to have more relevance in clinical practice in the future. Therefore, it becomes important to understand this condition, to estimate prognostic factors and to determine the intensity of local and systemic treatment.

## 2. Case Report

The patient is female, 60 y, with no comorbidities, with a 2-month history of abdominal pain and rectal bleeding. She was submitted to proctosigmoidoscopy and computerized tomography (CT) scans of abdomen and pelvis that revealed an infiltrative lesion in anal canal and a right adnexal mass with no evidence of peritoneal carcinomatosis or lymph nodes enlargement. A transvaginal ultrasound confirmed a right complex adnexal mass with 22 × 15 cm. By this time, laboratory exams showed CA 125 = 240 U/mL (<35 U/mL) and other tumor markers within normal range. Biopsy from the anal canal lesion suggested a poorly differentiated squamous cell carcinoma (SCC), confirmed by immunohistochemistry (IHC) positive for p63. Pelvic magnetic resonance imaging (MRI) and endorectal ultrasound confirmed a T3N0 tumor (AJCC 7th edition, 2010).

Patient proceeded to a total hysterectomy, bilateral salpingooophorectomy and inspection of the abdominal cavity. Histology revealed a moderately differentiated ovarian serous adenocarcinoma in the 22 cm right adnexal mass and an involvement of the left ovary. Endometrium and omentum were free of disease (FIGO stage Ic). IHC was positive for cytokeratin 7 (CK 7) and Wilms tumor gene product (WT-1) and negative for cytokeratin 20 (CK 20), cancer antigen 125 (CA 125), and caudal-related homeobox gene 2 (CDX-2). After resection CA 125 level has decreased to normal range (7 U/mL). Concerning the anal canal cancer, chemoradiotherapy with fluorouracil and mitomycin (Nigro's regimen) was performed. She also received 6 cycles of adjuvant chemotherapy for ovarian tumor with carboplatin (area under curve, AUC 6) and paclitaxel (175 mg/m^2^), administered every 3 weeks.

**Figure 1:**
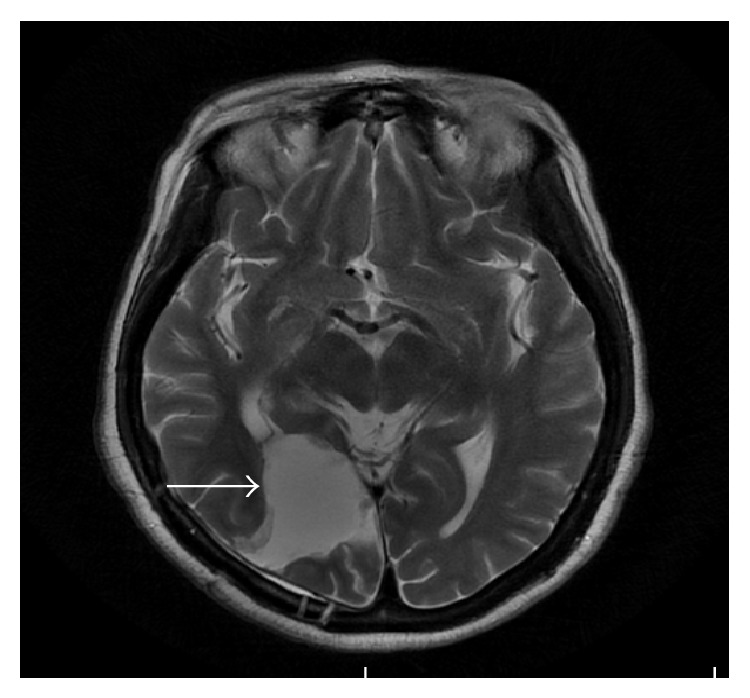
T2-weighted cranial MRI showed a 4.5 × 4.1 × 6.2-cm solitary BM (white arrow) in the right parietal-temporal-occipital lobe with intense contrast enhancement and a 0.8 cm displacement of midline structures. The lesion also compresses occipital horn of the lateral ventricle posteriorly and is surrounded by a wide area with alteration of signal that could correspond to edema or tumor infiltration.

Fifty-four months later, she was admitted in the hospital with decreased level of consciousness, left hemiparesis, and left homonymous hemianopia. Cranial MRI showed a 4.5 × 4.1 × 6.2 cm solitary BM in the right parietal-temporal-occipital lobe, with a wide perilesional edema and a 0.8 cm displacement of midline structures (Figure 1). Then, she proceeded to craniotomy and macroscopic debulking of the lesion. Histology revealed a completely excised poorly differentiated adenocarcinoma with extensive areas of necrosis and IHQ confirmed a metastatic ovarian serous adenocarcinoma (CK7, estrogen, and WT-1 positive, CK 20 negative). After surgery, she received whole brain radiotherapy (WBRT; 30 Gy in 10 fractions with 6 MV parallel opposed fields). No evidence of systemic disease was detected and further chemotherapy was not given.

Twenty-one months later, she developed headaches and mental confusion. New cranial MRI suggested a meningeal thickening and cerebrospinal fluid (CSF) analysis confirmed meningeal carcinomatosis. At this moment, contrast-enhanced CT scans of thorax, abdomen, and pelvis did not reveal any systemic recurrence and CA-125 level was normal. Patient was submitted to 3 infusions of intrathecal chemotherapy with methotrexate 12 mg plus dexamethasone 12 mg/dosis/twice a week; however, no clinical improvement nor decrease of cell count in CSF was obtained and she died from progressive neurologic involvement two months later.

## 3. Discussion

Brain metastasis is a common condition in cancer patients and more than 170,000 new cases are diagnosed annually in US. Most common primary cancer are lung, breast, kidney and melanoma; however, female genital tract is a uncommon source, except for choriocarcinoma [12].

Nasu et al. analyzed retrospectively 139 women with gynecologic cancer with isolated BM treated in 15 Japanese centers, between 1995 and 2009. Fifty-six (40.3%) had ovarian cancer, fallopian tube or peritoneum cancer, 33 (58.9%) had a serous histology tumor, and 48 (85.7%) had a FIGO stages III-IV disease. The median survival time was 12.5 months for the ovarian cancer group, 6.2 months for the corpus cancer, and 5.0 months for cervical cancer [13].

The case presented here disclosed two important features: the late relapse in CNS (54 months) and the early stage disease at diagnosis (FIGO Ic).

The median interval between diagnosis of ovarian carcinoma and BM ranged from 0 to 133 months with a median time of 24.3 months [5]. In this case, the late relapse might be associated with an early stage of primary tumor, a moderately differentiated tumor, and the absence of extracranial disease.

Pakneshan et al. performed a systematic review which screened 20 series with 349 cases of CNSm from EOC and showed that only 59 (17%) had a FIGO stages I-II disease [14]. Furthermore, only 8 of 56 patients (14%) with CNSm from EOC reported by Nasu et al. had an early stage disease [13]. According to E. Piura and B. Piura, the interval between diagnosis of ovarian carcinoma and BM was five times longer in stage I/II than in stage III/IV EOC [5].

Patients with poorly differentiated serous ovarian adenocarcinoma (grade 3) have CNS relapse earlier than those with well/moderately differentiated ovarian cancer (grades 1 and 2) (1.5 versus 4.73 years, *P* value = 0.03) [7]. Moreover, women who had extra cranial metastasis at the time of initial diagnosis of ovarian cancer had a shorter time interval between diagnosis of EOC and mCNS (24.6 versus 61.7 months, *P* value = 0.040) [14].

Among those patients treated with platinum and taxane-based chemotherapy evaluated in retrospective series, relapse in CNS occurs about 22 months after diagnosis of primary cancer and survival usually is limited (Table 1).

More recently there have been attempts to assess the prognostic impact of clinical and pathologic variables to determine the intensity of local control and necessity of systemic treatment.

A higher Karnofsky performance scale score (KPS) and solitary BM (opposed to multiple lesions) have been consistently related to a longer survival time [4, 5, 7]. For example, Pakneshan et al. revealed a median survival time of 21.4 and 9.2 months for patients with solitary and multiple lesions, respectively [14].

In a multicenter retrospective analysis with 74 women with CNSm from EOC, Sehouli et al. verified that platinum sensitivity was associated with a more favorable outcome (HR 0.23, 95% CI 0.12–0.48). However, these findings could be explained by the negative impact of platinum resistance that overrules the effect of any treatment strategies in this subgroup of patients. Moreover, data concerning response to treatment modalities were not evaluable due to the nonrandomized and retrospective nature of the study [4].

Among patients who develop BM from ovarian cancer, 47% had isolated CNSm and 53% had concomitant extracranial disease [5]. The presence of systemic disease at the time of brain relapse has remained a controversial prognostic factor. Three retrospective series have found a negative impact in survival in those patients who presented extracranial disease when compared to those with solitary brain relapse [6, 7, 13]. However, Sehouli et al. did not confirm this negative impact in their analysis and patients with or without extracranial disease presented similar survival time (6.2 versus 6.3, *P* = 0.370) [4]. The CA-125 level was also evaluated as a possible prognostic factor. Anupol et al. found that 66% (10 of 15) of women diagnosed with CNSm had an elevated CA-125, but level of this tumor marker did not predict length of survival [6].

Because of the rarity of this metastatic site, the optimal management for CNSm from EOC is not well defined. As in other primary sites, physicians must take into account KPS and number of brain lesions to estimate the prognosis and determine the intensity of local and systemic treatment.

Conventionally, the recommended treatment includes debulking surgery followed by radiotherapy, and this strategy seems to result in better outcomes when compared to WBRT alone [15]. In Nasu et al., women who had been submitted to combined treatment (surgery plus WBRT) had a longer survival (median 23 months) than those submitted to WBRT or surgery alone (5.3 and 6.9 months) (*P* < 0.01), but it was a retrospective nonrandomized study liable to selection bias [13]. Furthermore, since there is no sufficient evidence to recommend chemotherapy in patients with isolated BM from EOC, this practice should be avoided out of clinical trial context.

## 4. Conclusion

Late CNSm can be a late manifestation in patients with early ovarian cancer who had received platin and taxane-based chemotherapy. Although there is no standard treatment, we favor resection followed by radiotherapy in isolated brain metastasis. There is no evidence of benefit in offering chemotherapy in those patients who had not extracranial disease.

## Figures and Tables

**Table 1:** Retrospective series that evaluated patients with CNSm from EOC.

Author, year (reference)	Regimen of chemotherapy^*^	Number of patients with CNSm	Median interval between diagnosis of EOC and CNSm in months (range)	Median survival after diagnosis of OC in months (range)	Median survival after diagnosis of CNS metastases in months (range)
Anupol et al., 2002 [6]	Cisplatin and paclitaxel	15	22 (0–53)	38 (9–82)	6 (0–49)
Cohen et al., 2004 [7]	Cisplatin and paclitaxel	72	22 (0–219)	40.5 (21.5–60)	6.3 (5–8)
Chen et al., 2011 [8]	Carboplatin and paclitaxel	10	24.3 (7–55)	NA	3 (0–16)
Kumar et al., 2003 [9]	Carboplatin and paclitaxel	18	29 (0–101)	30.5 (5–110)	4 (1–74)
Tay and Ajesh, 2005 [10]	Carboplatin and paclitaxel	4	16.5 (8–65)	NA	19,5
Kastritis et al., 2006 [11]	Carboplatin and paclitaxel	8	17,2	67 (48–85)	22 (12–34)

^*^Adjuvant, palliative, or neoadjuvant chemotherapy received before diagnosis of CNSm.
